# Prognostic significance of PTOV1 expression in cancers: A protocol for systematic review and meta-analysis

**DOI:** 10.1097/MD.0000000000028149

**Published:** 2021-12-17

**Authors:** Yue Yang, Nan Li, Guangwei Tian

**Affiliations:** a Department of Pathology, the Third Affiliated Hospital of Jinzhou Medical University in Jinzhou, Liaoning, China; b Department of Radiation Oncology, the First Affiliated Hospital of China Medical University in Shenyang, Liaoning, China.

**Keywords:** cancer, meta-analysis, prognosis, protocol, PTOV1

## Abstract

**Background:**

Prostate tumor overexpressed-1 (PTOV1) was firstly depicted as gene and protein overexpressed in prostate cancers and preneoplastic lesions of high-grad intraepithelial neoplasia. Recently, people have paid recent attention to the oncogenic PTOV1 protein as a regulator with various cellular functions and pathways that tend to enhance cell growth and self-renewal in numerous cancer cell types. Its prognostic role in cancers remains controversial.

**Methods:**

Eligible studies are identified by comprehensively searching literature in all available databases. The associations between PTOV1 expression and overall survival, disease-free survival, relapse-free survival, progression-free survival, and clinicopathological characteristics are estimated by employing hazard ratios and the confidence intervals of 95%. STATA 12.0 software was adopted to perform the meta-analysis.

**Results:**

This study will provide high-quality synthesis to evaluate the associations between PTOV1 expression and overall survival, disease-free survival /relapse-free survival , progression-free survival, and clinicopathological features.

**Conclusion:**

The study will provide updated evidence to assess whether the expression of PTOV1 is in association with poor prognosis in patients with cancers.

**PROSPERO registration number:**

CRD42020183853.

## 1 Introduction

Prostate tumor overexpressed-1 (PTOV1) is a 46 kDa protein with a tandem duplication of 2 repeated homology blocks of the sequence of 151 and 147 amino acids closely related to each other, located on the 19q 13.3–13.4 chromosome.^[1]^ PTOV1 was first identified while screening the genes whose overexpression is made in prostate cancer, which is involved in prostate cancer progression.^[2,3]^ As an adaptor, PTOV1 is conserved in vertebrates (mammals and fish) and arthropods (insects). The protein interacts with a variety of factors in the nucleus and the cytoplasm, regulates gene expression at transcription and posttranscriptional levels, and promotes proliferation and motility of cancer cells. PTOV1 is overexpressed in NSCLC cell lines and tissues. Although PTOV1 increased cell apoptosis, inhibited cell migration and invasion.^[4]^ Overexpression of PTOV1 suggests poor prognosis in hepatocellular carcinoma(HCC),^[5]^ and nasopharyngeal carcinoma (NPC),^[6]^ cervical cancer,^[7]^ epithelial ovarian cancer.^[8]^ High expression of PTOV1was significantly associated with advanced TNM stage.^[9]^

In view of all these situations, there is paid new attention to reveal the prognostic value and clinicopathological association of PTOV1 abnormal expression in cancers.

## 2 Methods

### 2.1 Study registration

The protocol of the systematic review has been registered on PROSPERO. The registration number is CRD42020183853. The meta-analysis protocol will be formulated following the Preferred Reporting Items for Systematic Reviews and Meta-Analysis Protocols (PRISMA-P) statement guidelines.^[10]^

### 2.2 Data sources and search strategy

Comprehensive literature retrieval was conducted in Pubmed, Embase, Cochrane Central Register of Controlled Trials (CENTRAL), China National Knowledge Infrastructure Database (CNKI), Chinese Scientific Periodical Database (VIP Database), and WanFang Database from the beginning to December 2020. The strategy will be developed based on a discussion by all reviewers, in accordance with the Cochrane handbook guidelines. The following search terms will be used: “tumor,” “cancer,” “neoplasm,” “carcinomas,” “PTOV1,” “prognostic,” “survival,” “outcome,” and “prognostic biomarker.” The preliminary search strategy in Table 1 will be used for Pubmed. This retrieval strategy will be modified and adjusted according to the specific requirements of other databases.

**Table 1:** Preliminary search strategy PubMed.

No.	Search items
#1	((((((((“Neoplasms”[Mesh]) OR (neoplasms[Title/Abstract])) OR (neoplasm[Title/Abstract])) OR (cancers[Title/Abstract])) OR (cancer[Title/Abstract])) OR (tumors[Title/Abstract])) OR (tumor[Title/Abstract])) OR (carcinomas[Title/Abstract])) OR (carcinoma[Title/Abstract])
#2	((“PTOV1 protein, human” [Supplementary Concept]) OR (“PTOV1”[Title/Abstract])) OR (“prostate tumor over expressed gene 1 ”[Title/Abstract])
#3	((((((“Prognosis”[Mesh]) OR (“prognosis”[Title/Abstract])) OR (“outcome”[Title/Abstract])) OR (“prognostic value”[Title/Abstract])) OR (“survival”[Title/Abstract])) OR (“prognostic biomarkers”[Title/Abstract])) OR (“prognostic biomarker”[Title/Abstract])
#4	#1and#2and#3and#4

### 2.3 Inclusion criteria for study selection

1. Patients diagnosed with cancer by pathology and histology;
2. PTOV-1 expression detected in primary tumor tissues;
3. Full text, original Chinese, and English Reseach papers;
4. Statistical results, including hazard ratios (HRs) and 95% confidence intervals (CIs), directly reported or calculated from demographic data or survival curves;

Reviews, case reports, conference abstracts, specialist experience, comments, and cell or animal studies are not included.

### 2.4 Data collection and analysis

#### 2.4.1 Selection of studies

**Figure 1:**
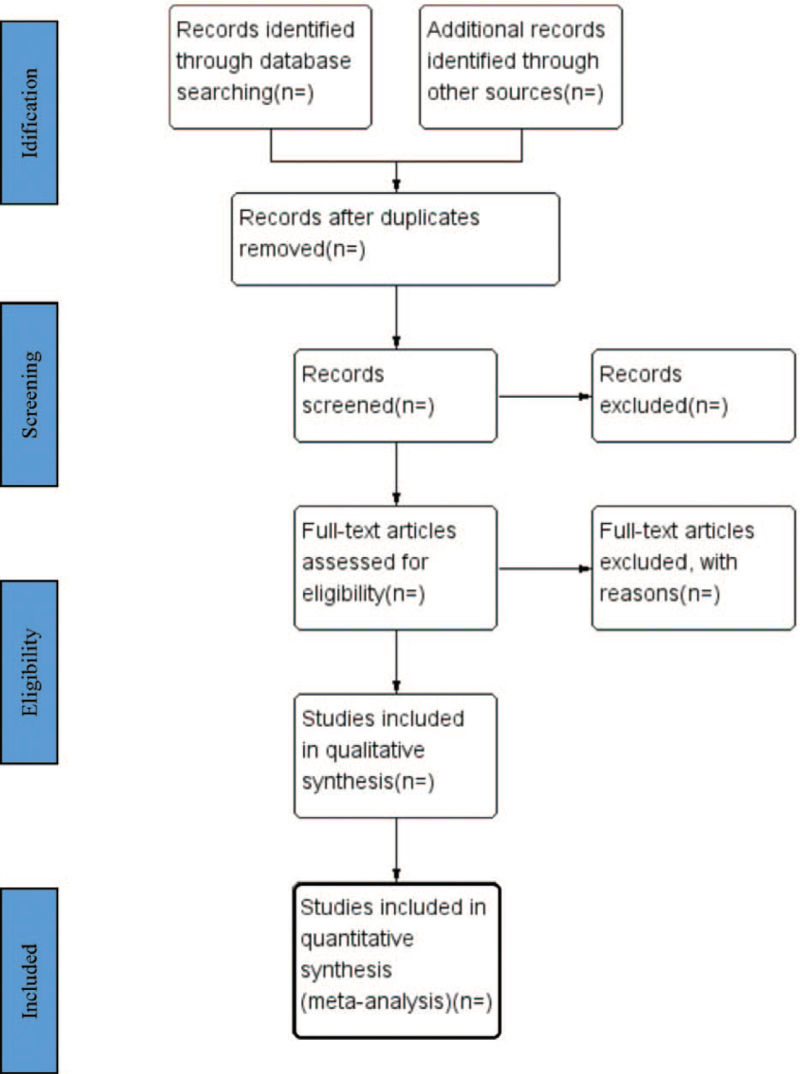
Flow diagram of study selection process.

Two researchers will search and screen independently. Before screening studies, all researchers have received evidence-based training. The results will be exported to the Endnote X8. It will delete duplicate documents. First, we will screen and evaluate the titles, abstract, and keywords of studies, and select those likely to be of relevance to our systematic review. Second, remaining full texts will be examined. The differences between the 2 researchers will be resolved by consensus or by a third independent arbitrator. The selection process will be shown in a PRISMA flow diagram (Fig. 1).

#### 2.4.2 Data extraction and management

The extraction of all data will be made by 2 investigators independently, and any difference will be solved by discussion. The information extracted includes name of the first author, publication time, country, number of patients, recruitment time, follow-up duration, analysis method, tumor type, clinicopathological features, method and score for its evaluation, PTOV1 detection method, positive expression rate, HRs, and their 95% CIs. For the studies that have not directly reported HRs and 95% CI, we will calculate or estimate them according to the method of Tierney from survival curves, which were constructed by the Kaplan-Meier method and log-rank test.^[11,12]^

### 2.5 Quality assessment in included researches

The quality assessment of the selected studies was employed the Newcastle–Ottawa Quality Assessment Scale (NOS). The NOS included 3 main aspects: selection, comparability, and outcome.^[13]^ Each research with the scores of at least 5 is regarded as high quality.^[14]^ In case of any dispute on quality assessment, the investigators shall resolve it through discussion or come to an agreement by an arbiter.

### 2.6 Measures of prognosis

Prognostic results include the whole survival, disease-free survival/relapse-free survival, and progression-free survival. The outcomes will be described as the HRs along with the 95% CIs or *P* values.

### 2.7 Management of missing data

If the inadequate and missing data are detected involved in trials, we will try to contact the corresponding author via email to integrate the data. In case of any failure, available data will be analyzed to perform the result.

### 2.8 Evaluation on heterogeneity

Cochran *Q* test and Higgins *I*^2^ statistics were used for heterogeneity test in all researches.^[15]^ When *I*^2^ ≥50% or *P* < .05, the result indicates heterogeneity, whereas *I*^2^ < 50% will be consider no heterogeneity. In cases of significant heterogeneity, we will explore the possible causes by sensitivity analysis and subgroup analyses.

### 2.9 Assessment of publication biases

If >10 studies are included, we will use funnel plots to assess Publication bias researches. The funnel plot visual inspection and statistical tests (Beg and Egger tests)^[16]^ will be used to assess publication bias.

### 2.10 Statistical analysis

STATA software (version 12.0; Stata Corp, College Station, TX) will be applied to perform the meta-analysis. The HRs and the corresponding 95% CIs are applied to perform the prognosis results. The fixed-effect model will be used for pooling homogeneous data (*I*^2^ < 50%). Otherwise, The fixed-effect model will be used for pooling homogeneous data (*I*^2^ < 50%).^[17]^ In addition, the sensitivity analysis and subgroup analysis will be used to investigate the causes of heterogeneity. *P* < .05 is regarded statistically.

### 2.11 Subgroup analysis

In cases of high heterogeneity, the applicable elements that may influence the outcomes will be determined by conducting subgroup meta-analyses. Different countries, type of cancers and statistical analyses will be regarded as subgroup analysis.

### 2.12 Sensitivity analysis

The “metaninf” STATA command (continuing exclusion of each research pooled HRs) will be used to conduct the sensitivity analysis, so as to investigate the robustness of the outcomes pooled.

## 3 Discussion

As a major public health issue in the world, cancer is the second major death cause in the United States.^[18]^ It is expected that cancer will rank as the major death cause and the most key limit to the increase in average lifetime in the world in the 21st century. In accordance with the estimates from the World Health Organization (WHO) in 2015, as the first or second major cause of death before 70 years old in 91 of 172 countries, cancer occupies the 3^rd^ or 4^th^ place in an extra 22 countries.^[19]^

Firstly, PTOV1 was depicted as gene and protein whose overexpression is made in prostate tumors and preneoplastic lesions of high-level intraepithelial neoplasia.^[20]^ According to PTOV1, double functions in regulating gene expression at the levels of transcription and translation are showed. As an element of transcription, it is observed that PTOV1 is related to the regions regulated of at least three genes which modulate transcription by recruiting deacetylases and other DNA modifying enzymes. The reports of the relationship with transcriptional repressor complexes, such as some HDACs and NCoR were made for repressing a few genes (*HES1, HEY1*, and *DKK1*).^[21]^

This review has several thresholds. Caused by language barrier, researches published in English and Chinese are only provided, which may bring heterogeneity risk. Besides, there are different approaches and cut-off definitions for assessing PTOV1 expression.

## Author contributions

**Conceptualization:** Yue Yang and Guangwei Tian

**Data collection:** Yue Yang

**Formal analysis:** Nan Li.

**Funding acquisition:** Guangwei Tian, Nan Li.

**Resources:** Guangwei Tian and Nan Li

**Software:** Guangwei Tian, Nan Li.

**Supervision:** Yue Yang and Guangwei Tian

**Writing – original draft:** Yue Yang and Guangwei Tian

**Writing – review & editing:** Yue Yang and Guangwei Tian
